# Long-term mercury contamination does not affect the microbial gene potential for C and N cycling in soils but enhances detoxification gene abundance

**DOI:** 10.3389/fmicb.2022.1034138

**Published:** 2022-10-05

**Authors:** Beat Frey, Basil M. Rast, Weihong Qi, Beat Stierli, Ivano Brunner

**Affiliations:** ^1^Forest Soils and Biogeochemistry, Swiss Federal Institute for Forest, Snow and Landscape Research WSL, Birmensdorf, Switzerland; ^2^FGCZ Functional Genomics Center Zurich, ETH Zürich and University of Zürich, Zürich, Switzerland; ^3^SIB Swiss Institute of Bioinformatics, Geneva, Switzerland

**Keywords:** shotgun metagenomics, mercury, *mer* genes, *hgcAB* genes, biogeochemical cycling, CAZy (carbohydrate-active enzymes), EggNOG, soil

## Abstract

Soil microorganisms are key transformers of mercury (Hg), a toxic and widespread pollutant. It remains uncertain, however, how long-term exposure to Hg affects crucial microbial functions, such as litter decomposition and nitrogen cycling. Here, we used a metagenomic approach to investigate the state of soil functions in an agricultural floodplain contaminated with Hg for more than 80 years. We sampled soils along a gradient of Hg contamination (high, moderate, low). Hg concentrations at the highly contaminated site (36 mg kg^–1^ dry soil on average) were approximately 10 times higher than at the moderately contaminated site (3 mg kg^–1^ dry soil) and more than 100 times higher than at the site with low contamination (0.25 mg kg^–1^ dry soil; corresponding to the natural background concentration in Switzerland). The analysis of the CAZy and NCyc databases showed that carbon and nitrogen cycling was not strongly affected with high Hg concentrations, although a significant change in the beta-diversity of the predicted genes was observed. The only functional classes from the CAZy database that were significantly positively overrepresented under higher Hg concentrations were genes involved in pectin degradation, and from the NCyc database dissimilatory nitrate reduction and N-fixation. When comparing between low and high Hg concentrations the genes of the EggNOG functional category of inorganic ion transport and metabolism, two genes encoding Hg transport proteins and one gene involved in heavy metal transport detoxification were among those that were highly significantly overrepresented. A look at genes specifically involved in detoxification of Hg species, such as the *mer* and *hgc* genes, showed a significant overrepresentation when Hg contamination was increased. Normalized counts of these genes revealed a dominant role for the phylum *Proteobacteria*. In particular, most counts for almost all *mer* genes were found in *Betaproteobacteria*. In contrast, *hgc* genes were most abundant in *Desulfuromonadales*. Overall, we conclude from this metagenomic analysis that long-term exposure to high Hg triggers shifts in the functional beta-diversity of the predicted microbial genes, but we do not see a dramatic change or breakdown in functional capabilities, but rather functional redundancy.

## Introduction

Mercury (Hg) is a non-essential heavy metal with no known biological functions (Nies, 1999). It is a toxic pollutant found in all environments due to long-range atmospheric transport. Globally, it has been estimated that more than 7,500 Mg of Hg is emitted to the atmosphere every year, about one-third of which is derived from anthropogenic sources (Pirrone et al., 2010). Geogenic Hg emissions come mainly from volcanic eruptions, other geothermal activities, and from rock weathering, while anthropogenic Hg emissions come mainly from fossil-fuel-fired power plants, small-scale gold mining for gold amalgamation, non-ferrous metal manufacturing, cement production, and waste disposal (Pirrone et al., 2010). Industrial waste disposal in particular is a major source of Hg contamination, with approximately 187 Mg emitted per year (Pirrone et al., 2010).

In the soils, the toxicity of Hg is highly dependent on its chemical speciation. Mercury is transformed biotically and abiotically into several major forms, including elemental Hg^0^, mercuric Hg^2+^, and methylmercury (MeHg), with MeHg being the most prevalent organo-Hg and a potent neurotoxin (Date et al., 2019). In particular, MeHg has a high affinity for sulfhydryl ligands in amino acids, leading to changes in protein structure and loss of function (Nies, 2003). Therefore, MeHg bioaccumulates in the food web, and humans are exposed to this neurotoxin through their diet, particularly by consuming Hg-contaminated animals Diez (2009).

*Bacteria* and *Archaea* possess various mechanisms to cope with high Hg concentrations in soils. The *mer* operon system in *Bacteria* and *Archaea* codes for the detoxification of proteins and is a known bacterial resistance system against Hg (Boyd and Barkay, 2012). The central gene for Hg resistance in the *mer* operon system is *merA*. This gene codes for the mercuric reductase enzyme, a flavoprotein located in the cytoplasm and using NADPH as electron donor; it catalyzes the conversion of Hg^2+^ to volatile Hg^0^ (Barkay et al., 2003). The conversion of Hg^2+^ to MeHg is mainly carried out by certain anaerobic *Bacteria* and *Archaea*. However, the physiological role of microbial MeHg production is unclear, as Hg methylation apparently does not confer resistance to Hg toxicity. Interestingly, several Hg methylators have been shown to simultaneously methylate Hg and demethylate MeHg (Date et al., 2019).

Bacterial species currently known to methylate Hg include *Desulfovibrio* spp. and *Geobacter* spp., and archaeal species known to methylate Hg include species of *Methanomicrobia* (Gilmour et al., 2018). In addition, some bacterial and archaeal species carry *hgcAB* genes and, thus, are suspected to produce MeHg as well (e.g., *Bacteroidetes*, *Chloroflexi*, *Nitrospirae*, Thermoplasmata; Gilmour et al., 2018). The gene *hgcA* encodes a corrinoid-dependent protein that presumably functions as part of a methyltransferase, and *hgcB* encodes an associated ferredoxin protein that potentially reduces the corrinoid center of *hgcA* (Gilmour et al., 2013). Both *hgcA* and *hgcB* occur in bacterial and archaeal taxa. However, the *hgcAB* gene pair is relatively rare, occurring in only ∼1.4% of sequenced microbial genomes (Podar et al., 2015). Nevertheless, microorganisms carrying these genes are distributed worldwide in highly diverse anaerobic settings, including soils, sediments, invertebrate digestive tracts, and various extreme environments. It is not known why microorganisms methylate Hg, but this process is generally not thought to be a Hg detoxification mechanism, as microorganisms harboring *hgcAB* genes are apparently no less susceptible to Hg toxicity than those lacking them (Gilmour et al., 2011; Cooper et al., 2020).

A recent review stated that exposure to Hg is a threat to microbial soil functions involved in C and N cycles (Durand et al., 2020). However, the majority of studies have been carried out shortly after Hg contamination (e.g., Frey and Rieder, 2013; Frossard et al., 2017). Thus, it remains uncertain whether crucial soil functions, such as litter decomposition and N cycling, are hampered after long periods of exposure to high Hg levels. Here, we aimed to investigate a Swiss agricultural floodplain soil, contaminated by the Hg waste of a chemical plant for about a decade, by studying the condition of soil functions using a metagenomic approach. The Hg contamination started in 1917, when Hg was used as a catalyst during the production of acetaldehyde, vinyl acetate and vinyl chloride, as well as in chlor-alkali electrolysis, and then released as chemical waste into a water channel (Osterwalder et al., 2019). The sediment of that channel was then regularly dredged from 1935 to 1975 and spread as fertilizer on agricultural soils or as fill material in settlement areas along the channel (ForumUmwelt, 2011). In 2012, the environmental agency of the canton of Valais required soil investigation to assess the extent of the polluted area, because the Hg concentrations drastically exceeded the critical limit of 0.5 mg kg^–1^ soil (VBBO, 1998). In Switzerland, soils with Hg contamination ≥20 mg kg^–1^ soil require disposal (Portmann et al., 2013). Since 2012, several scientific studies have been conducted to investigate chemical and physical parameters at the site (Gilli et al., 2018; Gygax et al., 2019; Osterwalder et al., 2019; Gfeller et al., 2021), as well as microbial parameters and communities (Frossard et al., 2018).

In an initial study we focused on the altered diversity of microbial communities under high Hg exposure over long periods (Frossard et al., 2018). Here, we focused on the functional gene potential of the microbial communities and how it is characterized in soils that are contaminated with Hg over many years. In particular for highly long-term contaminated soils, we hypothesized that: (1) general physiological processes assessed by the presence of genes from the EggNOG database are strongly modified, (2) C- and N-cycling processes assessed by the presence of genes of the CAZy and NCyc databases are strongly affected, (3) Hg detoxification processes assessed by the presence of *mer* and *hgc* genes are strongly influenced.

## Materials and methods

### Study site and soil samples

Soils were collected in October 2015 from a pasture in an agricultural floodplain near the town of Raron (CH; 46°18′10.6″N, 7°48′34.2″E), where high Hg contamination had been detected (ForumUmwelt, 2011). The Hg contamination originated from the sediment of a water channel (“Grossgrundkanal”) into which large quantities of Hg (estimated at 50–250 t) were discharged from 1917 on by a chemical plant located upstream. The sediment was then dredged and spread on the pastures between approximately 1935 and 1975 (Osterwalder et al., 2019). This resulted in a gradient of contamination, with the highest Hg concentration in soil occurring near the channel and decreasing concentrations with increasing distance from the channel. Soil samples were taken along this gradient at sites with different levels of Hg concentration (low contamination level at 100 m distance from the water channel, moderate contamination level at 30 m distance, high contamination level at 5 m distance). Four replicated samples, positioned 20 m apart from each other, were used for each of the Hg levels, resulting in a total of 12 soil samples (see also Frossard et al., 2018). Soils were collected from a depth of 0–10 cm (A horizon) using a soil corer with a diameter of 7 cm. The fresh soil samples were mixed and sieved (2 mm) and then split for storage, with one subsample kept at 4°C in the dark for one week for chemical, physical and biological analysis and the other at −20°C for DNA extraction.

### Soil physico-chemical parameters

Soil samples were dried overnight at 105°C to measure their gravimetric water content. Soil texture was determined with the hydrometer technique according to Gee and Bauder (1986). The soil pH was measured in ultrapure water with a soil to water ratio of 1:2 using a glass electrode linked to a pH meter (FEP20-FiveEasy Plus, Mettler-Toledo GmbH, Switzerland). Around 2 g of well-homogenized soil was milled with a Teflon ball mill, and around 40 mg of soil was subsequently weighed into tin caps for measurement of the total C (TC) and total N (TN) concentrations with a CN elemental analyzer (NC2500, CE Instruments, Italy). Organic C (C_org_) was separated from inorganic C and was quantified according to Walthert et al. (2010). Water extractable Hg was extracted with milli-Q water for 16 h in a slurry at a ratio of 1:10 g soil ml^–1^ (Lazzaro et al., 2006; Rieder and Frey, 2013). Total Hg concentrations in soils were analyzed using a direct Hg analyzer (AMA 254 Mercury Analyzer, LECO Corporation, St. Joseph, MI, USA; detection limit 0.001 μg Hg g^–1^ dw), and water-extractable Hg concentrations were determined using an Inductively Coupled Plasma Mass Spectrometer (ICP-MS, 7700x, Agilent Technologies, Japan).

### Soil microbiological parameters

Basal respiration was measured in a closed soil-chamber system connected to a Li-8100 infrared gas analyzer (LI-COR Inc., Lincoln, NE, USA). The soil containers were connected to the CO_2_ analyzer. CO_2_-free air flowed at a rate of about 0.16 L min^–1^ through the containers, and entrained the CO_2_ just released from the soil to the infrared gas analyzer. After 13 d of incubation, the gas flow and CO_2_ concentration were recorded with three measurements within 6 h. The basal respiration was then calculated according to Rieder and Frey (2013) and the fluxes reported as μg CO_2_ d^–1^ g^–1^ dry soil. Potential nitrification rate was determined using the shaken slurry method (Hart et al., 1994). Nitrification potential was calculated by linear regression of accumulated nitrate over time and expressed as ng NO_3_^–^ h^–1^ g^–1^ dry soil (Frey et al., 2020).

### DNA extraction and relative abundances of taxonomic and functional genes

DNA was extracted from all twelve soil samples using the PowerSoil DNA Isolation Kit (Qiagen, Hilden, Germany) and was quantified using the high-sensitivity Qubit assay (Thermo Fisher Scientific, Reinach, Switzerland). The amount of DNA extracted from soils was used as a proxy for the microbial biomass, as a previous study has shown that the amount of DNA extracted from soils can be used as an approximation (Frey et al., 2022).

Relative abundances of the bacterial 16S rRNA gene, fungal ITS, *nifH* (nitrogen fixation), bacterial *amoA* and archaeal *amoA* (nitrification)*-*DNA copies were determined with quantitative real-time PCR (qPCR) on an ABI7500 Fast Real-Time PCR system (Applied Biosystems, Foster City, CA, USA) according to Frey et al. (2020). qPCR amplifications of Hg reductase (*merA*) gene copies were performed with the MerAF and MerAR primers (Larose et al., 2013). The initial DNA denaturation was conducted at 95°C for 15 min. Each of the following 40 amplification cycles involved a denaturation step at 95°C for 30 s, primer annealing at 60°C for 45 s and an extension phase for 45 s at 72°C. A final cycle included a denaturation step at 95°C for 15 s. Primer annealing was done at 60°C for 1 min followed by denaturation at 95°C for 15 s. qPCR analyses were performed using 2.5 ng DNA in a total volume of 25 μL containing 0.5 μm of each primer, 0.2 mg mL^–1^ of bovin serum albumin (BSA), and 12.5 μL of QuantiTect SYBR Green PCR master mix (Qiagen, Hirlen, Germany). Three standard curves per target region (correlations ≥0.997) were obtained using tenfold serial dilutions (10^–1^ to 10^–9^ copies) of plasmids generated from cloned targets (Frey et al., 2011). Data were converted to represent the average copy number of targets per g dry soil.

### Shotgun sequencing

Both library preparation and shotgun sequencing of eluted DNA of nine soil samples (three Hg levels, three replicates) were performed at Microsynth AG (Balgach, Switzerland). The canonical analysis of principal coordinates (CAP) of Frossard et al. (2018) indicated that the microbial community structures of the four replicates were very close to each other for each of the Hg contamination levels, which is why three instead of four replicates were considered sufficient for the metagenomic analysis. Library preparation was performed using the Illumina TruSeq DNA Library Prep Kit, and shotgun sequencing was performed using the Illumina NextSeq 2500 System (2 × 150 bp; Illumina Inc., San Diego, CA, USA). Raw sequences were deposited in the NCBI Sequence Read Archive under the accession number PRJNA794054.

### Metagenome assembly

Pre-processing of metagenomic reads, assembly of reads into contigs, contig binning, and functional and phylogenetic annotation of contigs and bins were achieved using a customized pipeline. Briefly, raw reads were quality checked using FastQC^1^. They were quality filtered and trimmed (i.e., pre-processed reads) using Trimmomatic v0.36 (*Q* = 20, minimum read length = 40; Bolger et al., 2014). Pre-processed read pairs were assembled into contigs (>200 bp) by iteratively building *de Bruijn* graphs using *k*-mers of increasing size with the *de novo* assembler MEGAHIT v1.1.3 (–k-min 27, –k-step 10; Li et al., 2015).

### Functional annotation and taxonomic classification

Protein-coding sequences contained in the assembled contigs were predicted with MetaGeneMark v3.38 (Zhu et al., 2010). To uncover the potential metabolic capabilities of the soil metagenomes, protein-coding genes were assigned to functions (i.e., functional genes). About 50% of the predicted genes were assigned to general metabolic and cellular functions using EggNOG v4.5 (evolutionary genealogy of genes: non-supervised orthologous groups), which classifies the genes into clusters of orthologous groups (COGs) of proteins and organizes the COGs into general functional categories (Jensen et al., 2008; Huerta-Cepas et al., 2016). Annotation to EggNOG v4.5 was performed using the EggNOG-mapper v1.0.3 with the DIAMOND search mode against all protein sequences (Huerta-Cepas et al., 2017). About 1% of the protein-coding genes were annotated to carbohydrate-active enzymes using the CAZy database (carbohydrate-active enzymes: release of July 2017 version; Cantarel et al., 2009). About 0.2% of the genes were annotated to N-cycling families using the NCyc database (syn. NCycDB: curated integrative database for fast and accurate metagenomic profiling of N-cycling genes; Tu et al., 2019). Annotations against the CAZy and NCyc databases were performed using SWORD v1.0.3 (Vaser et al., 2016) (−v 10^–6^; Anwar et al., 2019). In addition to the categorization by enzyme classes implemented in CAZy, a manual categorization of CAZy genes into different C substrates was performed as previously outlined (Perez-Mon et al., 2021; Frey et al., 2022).

Kaiju v1.7.4, a program for sensitive taxonomic classification of high-throughput sequencing reads from metagenomic whole genome sequencing (Menzel et al., 2016), was used for the taxonomic classification of the protein-coding genes using default settings and the prebuilt “nr_euk” database (version 2021-02-24) containing bacterial, archaeal, viral, fungal and microbial eukaryotic protein sequences from the NCBI BLAST non-redundant protein database. The helper program kaiju-addTaxonNames was utilized to convert NCBI taxon IDs to taxonomy.

### Identification of *mer* and *hgc* genes

The presence of *mer* and *hgc* genes among protein-coding sequences was determined by aligning the sequences against known *mer* and *hgc* sequences. Available nucleotide and protein sequences of *mer* and *hgc* genes were downloaded from NCBI protein and nucleotide database in December 2021 utilizing the Entrez Direct v15.4 (Kans, 2022). Protein-coding sequences were aligned against downloaded protein sequences using BLASTP 2.11.0+ (Camacho et al., 2009) with standard parameters. All sequences with an alignment that had an *e*-value <10^–6^ to a *mer* or *hgc* gene were considered a match.

For *merA*, also primer-specific protein-coding sequences were identified. The *merA* nucleotide sequences downloaded from NCBI were filtered for MerAF and MerAR (Larose et al., 2013) primer binding sites. Nucleotide sequences were blasted against a sequence consisting of the MerAF primer sequence, 50 “N” characters, and the reverse complement sequence of the MerAR primer using BLASTX v2.11.0+ with the following parameters: -task blastn -word_size 11 -dust no -evalue 1000. Nucleotide sequences were considered to contain a primer-binding site if they had a match with a maximum of two mismatches. Protein-coding sequences that aligned with any of the primer binding-site filtered nucleotide sequences were considered a primer-specific *merA* gene.

### Normalized counts of the abundance of protein-coding genes

Pre-processed read-pairs from each of the samples were mapped to the assembled contigs, using the BWA aligner v0.7.15 (bwa-mem; Li, 2013). The function “featureCounts” from the package Subread v1.5.1 (-minOverlap 10, Q = 10, -primary; Liao et al., 2014) was used to count the read-pairs that mapped to the assembled protein-coding gene sequences to obtain gene abundances.

### Statistical analyses

Results from all statistical tests performed in this study were considered significant at *P* < 0.05 unless indicated otherwise. Statistical significances of observed differences were assessed by applying factorial analyses of variance (ANOVA) with Fisher’s protected least significant difference using StatView (v5.0, SAS Institute, Cary, NC, United States), and by applying permutational analyses of variance (PERMANOVA) with 10^5^ permutations and Monte Carlo approximated *P*-value using PRIMER v7 (Clarke and Gorley, 2015). For all other analyses RStudio and R v4.1.0 (R Core Team, 2019) were utilized.

Read counts were normalized to the protein-coding gene length in kilobases (kb) for intra-sample comparisons between different genes. For inter-samples comparisons, counts were normalized using the scaling with ranked subsampling (SRS) method, as implemented in the function “SRS” of the R package SRS v02.2 (Beule and Karlovsky, 2020). Normalization methods were combined for simultaneous intra- and inter-sample comparisons, unless specified otherwise.

The function “diversity” of the R package *vegan* v2.5-7 (Oksanen et al., 2020) was used to calculate Shannon’s diversity index. The beta-diversity of samples was assessed on Bray–Curtis dissimilarity matrix, produced with function “vegdist” of the R package *vegan*. Classical multidimensional scaling (MDS) was performed using the function “cmdscale” of the R package *stats*.

Pairwise DESeq2 analyses, using non-normalized read counts as input and standard parameters, were used to determine differentially abundant genes for all possible combinations of Hg contamination levels (function “DESeq” of the R package *DESeq2* v1.26.0; Love et al., 2014). Genes with read counts <10 over all samples of pairwise comparison were excluded prior to analysis to speed up computation. The reported number of genes after DESeq2 filtering corresponds to the genes that passed the simple read count filtering just described. Genes were considered as significantly over- or underrepresented only for pairwise comparisons with an adjusted *P*-value <0.01; *P*-values were adjusted for multiple testing using the Benjamini–Hochberg method.

Additional R packages that were used for the analyses were: *tidyverse* v1.3.1 (Wickham et al., 2019), *data.table* v1.14.0 (Dowle et al., 2021), *readxl* v1.3.1 (Wickham et al., 2020), and *ggpubr* v0.4.0 (Kassambara, 2020).

## Results

### Soil and microbial properties varied only slightly with Hg contamination

Total Hg concentrations averaged 36,097 μg Hg kg^–1^ dry soil (= 36.1 mg Hg kg^–1^ dry soil) at the highly contaminated site closest to the channel, with values approximately 10-fold higher than at the moderately contaminated Hg site and 100-fold higher than at the site with low Hg contamination farthest from the channel (*P* < 0.001; Table 1). The Hg concentration at the high contamination site is about two times higher than the recommended clean-up level of 20,000 μg Hg kg^–1^ for Switzerland (Portmann et al., 2013), and the Hg concentration at the low contamination site (251 μg Hg kg^–1^ dry soil) corresponds approximately to the natural background level in Switzerland (Rieder et al., 2011). Concentrations of water-extractable Hg were relatively low compared with total Hg, because only about 0.2% of the total Hg from the highly and moderately contaminated sites was extractable with water and only about 0.6% from the low contaminated site. However, soluble Hg showed a decrease along the gradient similar to that observed for total Hg: values in the highly contaminated site were about 14 times higher than in the site with moderate contamination and about 44 times higher than in the site with low contamination (*P* = 0.002; Table 1).

**TABLE 1:** Mean values of soil chemical, physical and biological properties (± SE) for the three Hg contamination levels low, moderate, and high Hg (*n* = 4) (compare also with Frossard et al., 2018).

	Low Hg	Moderate Hg	High Hg	*P* ^+^
**Soil chemical properties:**				
Hg_tot_ (μg kg^–1^ soil)	251 (± 46.3)	3,019 (± 945)	36,097 (± 2,398)	** < 0.001**
Hg_water extractable_ (μg kg^–1^ soil)	1.54 (± 0.35)	4.95 (± 1.61)	67.7 (± 17.5)	**0.002**
C_org_ (%)	1.63 (± 0.24)	2.76 (± 0.52)	2.18 (± 0.38)	0.19
C_tot_ (%)	2.71 (± 0.16)	3.78 (± 0.43)	3.88 (± 0.47)	0.10
N_tot_ (%)	0.17 (± 0.02)	0.29 (± 0.04)	0.31 (± 0.04)	**0.041**
C:N ratio	16.5 (± 1.16)	13.1 (± 0.33)	12.7 (± 0.57)	**0.013**
pH (H_2_O)	8.30 (± 0.10)	8.13 (± 0.11)	7.98 (± 0.04)	0.08
**Soil physical properties:**				
Sand (%)	44.4 (± 6.22)	22.8 (± 1.29)	29.4 (± 1.60)	**0.008**
Silt (%)	48.7 (± 6.03)	66.4 (± 2.16)	61.2 (± 1.00)	**0.023**
Clay (%)	7.0 (± 0.58)	10.8 (± 1.01)	9.5 (± 0.73)	**0.023**
**Soil biological properties:**				
Microbial biomass (μg DNA g^–1^ soil)	19.1 (± 2.57)	57.4 (± 7.98)	57.7 (± 8.65)	**0.005**
Basal respiration (μg CO_2_ d^–1^g^–1^ soil)	9.9 (± 1.81)	15.0 (± 2.79)	19.1 (± 2.92)	0.09
Nitrification rate (ng NO_3_ h^–1^ g^–1^ soil)	68.0 (± 2.91)	69.6 (± 3.06)	68.8 (± 1.61)	0.91
Abundance of gene copies (assessed by qPCR)*:				
*16S* (x10^10^) (g^–1^ soil)	2.85 (± 0.44)	5.61 (± 0.91)	4.92 (± 0.47)	**0.035**
*ITS* (x10^8^) (g^–1^ soil)	0.75 (± 0.14)	2.23 (± 0.85)	2.89 (± 1.10)	0.21
*nifH* (x10^8^) (g^–1^ soil)	2.52 (± 0.67)	8.50 (± 1.55)	8.53 (± 2.53)	0.06
*amoA Bacteria* (x10^7^) (g^–1^ soil)	0.37 (± 0.27)	1.42 (± 0.67)	1.48 (± 0.45)	0.25
*amoA Archaea* (x10^7^) (g^–1^ soil)	2.50 (± 0.46)	4.04 (± 0.81)	2.75 (± 0.41)	0.20
*merA* (x10^7^) (g^–1^ soil)	0.71 (± 0.17)	1.52 (± 0.34)	2.45 (± 0.24)	**0.004**

^+^Effect of Hg contamination assessed by analysis of variance (ANOVA); significant values (*P* < 0.05) are in bold. *16S: bacterial *16S* gene, *ITS*: fungal ITS gene, *nifH*: nitrogenase iron protein gene, *amoA*: ammonia monooxygenase subunit A gene, *merA*: mercuric reductase gene.

Soil pH decreased slightly across the sampled gradient from low to high Hg contamination, but differences were not significant (Table 1). Total C and N concentrations both increased toward the water channel (increasing Hg contamination level) but only N_tot_ increased significantly (*P* = 0.041), resulting in a significant decrease in the C:N ratio (*P* = 0.013). Similarly, the soil texture changed significantly along the gradient: the percentage of sand decreased (*P* = 0.008), while that of silt (*P* = 0.023) and clay increased (*P* = 0.023) with decreasing distance to the channel (higher Hg contamination; Table 1).

Microbial biomass, measured as μg DNA g^–1^ soil, was significantly enhanced with moderate and high Hg contamination levels (*P* = 0.005; Table 1). Basal respiration and nitrification rate, measured as CO_2_ and NO_3_ emissions from soils, respectively, tended to increase at the high Hg contamination level, but no significant differences were observed among the sites (Table 1). Overall, the number of 16S rRNA gene copies increased significantly (*P* = 0.035) with increasing Hg contamination level, whereas the number of ITS gene copies did not change significantly (Table 1). Genes involved in the N cycle, such as N-fixation genes (*nifH*) and ammonia-oxidizing genes (*amoA*) were not significantly affected by increasing Hg contamination level. In contrast, Hg reductase genes (*merA*) were significantly enhanced with increasing Hg contamination levels (*P* = 0.004; Table 1; compare also values in Frossard et al., 2018).

### Metagenomic sequencing

Metagenome sequencing of triplicate soil samples from the three Hg contamination levels (low, moderate, high) yielded, on average, 86 million raw reads per sample (Table 2). The total number of assembled reads into contigs was 17.7 × 10^6^, with a total size of 12.4 × 10^9^ base pairs (bp) (Supplementary Table 1). Contig length ranged from 200 bp to 207,300 bp with a N_50_ of 782 bp. Using MetaGeneMark, a total of 26.2 × 10^6^ genes were predicted to be present in the assembly.

**TABLE 2:** Mean number of sequences and percentage of protein-coding genes (CDS genes), and the relative abundance of CDS assigned to taxa at the domain level for the three Hg contamination levels.

	Low Hg	Moderate Hg	High Hg	*P* ^+^
Raw reads (× 10^6^)	99.2	85.8	82.7	0.34
High-quality reads (× 10^6^)	97.2	84.1	81.1	0.34
Reads aligned to contigs (%)	77.5	72.8	72.1	0.05
Aligned reads mapped to CDS genes (%)	78.6	77.1	77.6	0.24
*Bacteria* (%)	58.7	58.2	57.6	0.07
*Archaea* (%)	0.90	0.81	0.89	0.78
*Eukarya* (%)	0.23	0.23	0.23	0.54
Viruses (%)	0.03	0.03	0.03	0.91
Unclassified (%)	40.1	40.8	41.2	**0.04**

^+^Effect of Hg contamination assessed by analysis of variance (ANOVA); significant values (*P* < 0.05) are in bold.

Approximately 74% of the raw reads per sample were aligned to contigs, and about 78% of the aligned reads were mapped to protein-coding genes (CDS genes; Table 2). About 58% of the genes were assigned to bacterial taxa, whereas less than 1% were assigned to *Archaea*, *Eukarya* or Viruses. Around 40% of the genes remained unclassified. All these parameters remained unchanged across the Hg contamination levels, except the unclassified group, which significantly increased with increasing Hg level (*P* = 0.04; Table 2).

The number of predicted genes annotated against the EggNOG database was 12.5 × 10^6^ (47.5%), whereas the number of predicted genes annotated against the CAZy and NCyc databases was considerably smaller at 262.2 × 10^3^ (1.00%) and 42.9 × 10^3^ (0.16%), respectively (Supplementary Table 1).

### Diversity and differential abundance of predicted genes changed with Hg levels

**FIGURE 1:**
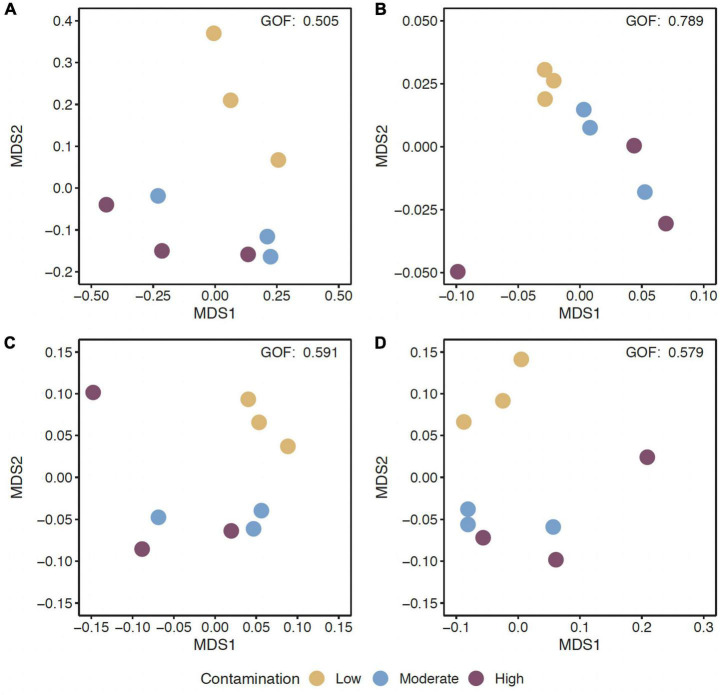
Non-metric multidimensional scaling (MDS) ordination of functional beta-diversity of all predicted genes **(A)** and of genes of the EggNOG database **(B)**, the CAZy database **(C)**, and the NCyc database **(D)** at sites with the three Hg contamination levels (low, moderate, high).

The alpha-diversity of predicted genes and for genes of the EggNOG, CAZy and NCyc databases, as expressed by richness and the Shannon index, was between 11.6 and 12.5 × 10^6^ genes (Supplementary Table 2), and the Shannon index was between 15.6 and 15.8, but both were not significantly different across the three Hg-contamination levels (Supplementary Table 3). In contrast, beta-diversity changed significantly with Hg contamination levels for all predicted genes, as well as for genes annotated against the EggNOG, CAZy, or NCyc database (*P* = 0.011 to 0.016; Supplementary Table 3). Pairwise comparisons of the Hg contamination levels revealed significant differences between low Hg and moderate Hg for all predicted genes and for the genes annotated against all three databases (*P* = 0.021 to 0.041; Table 3). Significant differences between low Hg and high Hg were present for all predicted genes (*P* = 0.041) and for the genes annotated against the NCyc database (*P* = 0.042). No significant differences were present between moderate Hg and high Hg (Table 3). Non-metric multidimensional scaling (MDS) ordination of functional beta-diversity of all predicted genes and of genes annotated against the three databases showed a clear separation of the samples from the low Hg contamination levels from the samples from the moderate Hg and high Hg levels, with goodness of fit (GOF) values between 0.505 and 0.789 (Figure 1).

**TABLE 3:** Pairwise comparison of the functional gene beta-diversity of all predicted genes and of the genes annotated with the EggNOG, CAZy, and NCyc databases of soils with different Hg contamination levels (low, moderate, high).

	All predicted genes		EggNOG		CAZy		NCyc	
Pairwise comparison*	*T* ^+^	*P*	*T*	*P*	*T*	*P*	*T*	*P*
Low Hg *vs.* moderate Hg	2.03	**0.035**	1.85	**0.041**	2.09	**0.021**	2.12	**0.037**
Low Hg *vs.* high Hg	1.97	**0.041**	1.73	0.05	1.84	0.06	1.94	**0.042**
Moderate Hg *vs.* high Hg	1.55	0.11	1.30	0.12	1.51	0.11	1.48	0.12

*Pairwise permutational multivariate analysis of variance (PERMANOVA) test. ^+^Values represent the *T*-value (*T*) and the level of significance (*P*); significant values (*P* < 0.05) are in bold.

To investigate changes in the abundance of functional genes with the Hg contamination we calculated log_2_-fold changes for the genes annotated with the EggNOG, CAZy, and NCyc databases (Table 4). The genes annotated against the EggNOG, CAZy and the NCyc databases showed the largest number of over- and underrepresented genes in the comparison between the low Hg and the high Hg contamination levels (between 16 and 127). In contrast, the comparison between moderate Hg and high Hg showed the smallest number of over- and underrepresented genes for the genes annotated against the three databases (between 0 and 15; Table 4). Total counts were 61,591 for EggNOG, 39,632 for CAZy, and 8,210 for NCyc database.

**TABLE 4:** Pairwise comparison and number of significantly (*P* < 0.01) over- and underrepresented differentially abundant genes annotated with the EggNOG, CAZy, and NCyc databases of soils with different Hg contamination levels (low, moderate, high). Total counts: EggNOG 61,591, CAZy 39,632, and NCyc 8,210.

Pairwise comparison	EggNOG		CAZy		NCyc	
	Overrep.	Underrep.	Overrep.	Underrep.	Overrep.	Underrep.
Low Hg *vs.* moderate Hg	53	63	35	24	8	6
Low Hg *vs.* high Hg	103	103	127	87	31	16
Moderate Hg *vs.* high Hg	0	8	0	15	0	4

### C- and N-cycling genes are not or only slightly affected by Hg

The classification of the functional potential genes of the EggNOG database was conducted via COG analysis. The results are summarized into four clusters (I–IV): information storage and processing (I); cellular processes and signaling (II); metabolism (III); and poorly characterized function (IV). The dominant known functions (> 1.2 × 10^6^ counts) among the 23 categories were replication, combination and repair (category L), and translation and ribosomal structure (category J) of cluster I, and energy production and conversion (category C), amino acid transport and metabolism (category E), and inorganic ion transport and metabolism (category P) of cluster III (Table 5). Overall, an increase in Hg contamination level had a significant positive effect on eight functional categories, as indicated by an over-represented number of genes. These functional categories were mainly genes of cluster II, such as those involved in cell wall/membrane/envelope biogenesis (category M; *P* < 0.001) and in intracellular trafficking, secretion, and vesicular transport (category U; *P* = 0.007), or genes of cluster III, such as those involved in coenzyme transport and metabolism (category H; *P* = 0.019) and in inorganic ion transport and metabolism (category P; *P* = 0.028; Table 5).

**TABLE 5:** Normalized counts of genes of the EggNOG database of soils with the three different Hg contamination levels (*n* = 3).

Functional categories	Low Hg	Moderate Hg	High Hg	*P* ^+^
**I) Information storage and processing:**				
RNA processing and modification (A)	2,960	3,070	3,037	0.77
Chromatin structure and dynamics (B)	1,821	1,969	1,839	0.62
Translation, ribosomal structure and b. (J)	1,508,524	1,560,644	1,639,956	**0.028**
Transcription (K)	1,206,550	1,213,370	1,259,839	0.23
Replication, combination and repair (L)	1,677,359	1,752,886	1,752,902	0.10
**II) Cellular processes and signaling:**				
Cell cycle control, cell division, chrom. (D)	191,131	199,463	207,096	**0.042**
Cell wall/membrane/envelope biogen. (M)	1,115,342	1,172,191	1,161,652	** < 0.001**
Cell motility (N)	83,656	89,462	94,148	0.30
Posttranslational modification, prot. (O)	792,763	816,456	826,600	**0.045**
Signal transduction mechanisms (T)	752,226	763,031	765,452	0.40
Intracellular trafficking, secretion, and v. (U)	347,385	359,107	381,949	**0.007**
Defense mechanisms (V)	396,746	382,594	387,756	0.77
Extracellular structures (W)	822	673	720	0.40
Nuclear structure (Y)	13	21	21	0.80
Cytoskeleton (Z)	3,171	3,220	3,303	0.86
**III) Metabolism:**				
Energy production and conversion (C)	1,652,084	1,760,398	1,779,031	0.09
Amino acid transport and metabolism (E)	1,228,039	1,269,264	1,280,520	0.17
Nucleotide transport and metabolism (F)	514,332	532,611	549,313	0.06
Carbohydrate transport and metabolism (G)	911,058	957,628	973,565	0.08
Coenzyme transport and metabolism (H)	656,485	689,321	686,603	**0.019**
Lipid transport and metabolism (I)	421,199	442,271	431,250	**0.040**
Inorganic ion transport and metabolism (P)	1,210,716	1,269,580	1,340,935	**0.028**
Secondary metabolites biosynthesis, tr. (Q)	192,976	197,890	196,003	0.61
**IV) Poorly characterized:**				
Function unknown (S)	6,505,870	6,634,080	6,617,739	**0.024**

^+^Effect of Hg contamination assessed by analysis of variance (ANOVA); significant values (*P* < 0.05) are in bold.

The most abundant genes of the CAZy database were those involved in anabolic processes, and for the NCyc database genes involved in organic degradation and synthesis (Ods; Table 6). The only functional types that were positively significantly influenced by the increasing Hg contamination levels were those involved in pectin degradation for the CAZy database, and in dissimilatory nitrate reduction (Dnr) and N fixation (Nif) for the NCyc database (Table 6).

**TABLE 6:** Normalized counts of genes of the CAZy and NCyc databases of soils with the three different Hg contamination levels (*n* = 3).

Functional types	Low Hg	Moderate Hg	High Hg	*P* ^+^
**CAZy genes:**				
Anabolic processes	511,500	514,877	501,455	0.15
Cellulose	78,901	78,574	77,745	0.91
Chitin	111,756	108,830	108,018	0.06
Hemicellulose	49,114	52,270	52,532	0.16
Lignin	38,038	37,009	39,232	0.16
Multiple	79,202	79,781	79,042	0.81
Murein	29,104	29,334	29,383	0.87
Oligosaccharides	143,112	149,743	147,832	0.06
Pectin	46,743	49,564	49,414	**0.018**
Starch	56,922	56,278	61,245	0.18
Unknown/others	93,081	93,841	90,784	0.71
**NCyc genes:**				
Anammox (Ana)	85	63	74	0.43
Assimilatory nitrate reduction (Anr)	11,528	12,266	11,963	0.30
Denitrification (Den)	12,332	12,623	14,335	0.08
Dissimilatory nitrate reduction (Dnr)	12,163	13,585	13,789	**0.021**
Den or Dnr	9,034	10,662	10,893	0.13
Nitrification (Nit)	1,692	1,699	1.776	0.97
Nitrogen fixation (Nif)	188	596	890	**0.012**
Organic degradation/synthesis (Ods)	136,475	137,641	141,614	0.20
Other	287	324	365	0.41

^+^Effect of Hg contamination assessed by analysis of variance (ANOVA); significant values (*P* < 0.05) are in bold.

**FIGURE 2:**
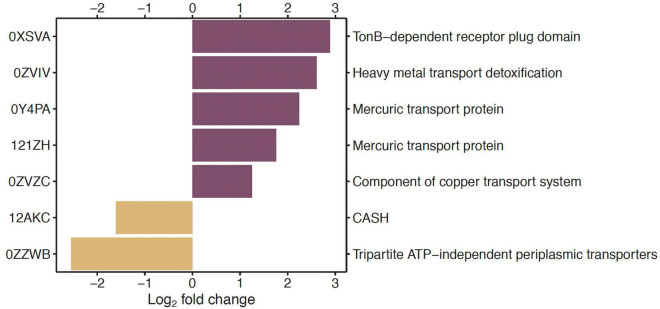
Under- and overrepresented genes in the pairwise comparison of low *vs.* high Hg contamination level, for genes annotated against the EggNOG database in the functional category of inorganic ion transport and metabolism (P). On the left side are the gene identification numbers, and on the right side are the corresponding functional genes. Only significantly (*P* < 0.01) differentially abundant genes between the two Hg contamination levels whose log_2_-fold change was lower than –1 or higher than +1 are displayed.

In the comparisons of genes between low Hg and high Hg levels for genes of the EggNOG functional category P (inorganic ion transport and metabolism), two genes coding for Hg transport proteins (0Y4PA, 121ZH) and one gene for heavy metal transport detoxification (0ZVIV) were among those that were highly significantly overrepresented (*P* < 0.01) with a log_2_ fold change >1.0 (Figure 2).

### Hg enhances detoxification genes and alters its abundance in proteobacterial taxa

Some of the *mer* and *hgc* genes involved in detoxification of the Hg ions showed a significant increase with increasing Hg contamination levels. In particular, these were genes responsible for regulation of the *mer* operon (*merD*), for Hg binding (*merP*), for Hg transport across membranes (*merC, merF, mer T*), and for Hg reduction (*merA* primer specific; Table 7). In addition, the *hgcB* gene, involved in the methylation of Hg to MeHg, was significantly enhanced, as was the *merB* gene, involved in the lysis of MeHg (Table 7). The schematic pathway of the Hg^2+^ and the reduction to Hg^0^ in the bacterial cell is given in the Supplementary Figure 1.

**TABLE 7:** Normalized counts of *mer* and *hgc* genes of soils with the three different Hg contamination levels (n = 3).

Genes	Coded protein*	Low Hg	Moderate Hg	High Hg	*P* ^+^
***mer* genes:**					
*merA*	Mercuric ion reductase	49,459	50,681	55,929	0.05
*merA* primer-specific^#^		5,790	5,773	7,250	**0.013**
*merB*	Organomercurial lyase	7,654	7,958	8,891	** < 0.001**
*merC*	Mercuric ion transport protein	14,230	14,889	18,634	**0.014**
*merD*	Regulator protein, repression of *mer* operon	3,402	3,103	4,568	**0.041**
*merE*	Methylmercury transport protein	656	580	623	0.63
*merF*	Mercuric ion transport protein	6,832	7,021	9,743	**0.037**
*merG*	Phenylmercury resistance protein	3,817	4,085	3.962	0.70
*merP*	Periplasmatic mercuric ion binding protein	16,266	16,253	20,523	**0.036**
*merR*	Regulator protein, activation of *mer* operon	66,768	65,615	71,758	0.16
*merT*	Mercuric ion transport protein	7,269	7,148	11,290	**0.025**
***hgc* genes:**					
*hgcA*	Transmembrane corrinoid-binding protein	135	313	211	0.09
*hgcB*	Ferredoxin-like protein	66	178	146	**0.036**

*According to Dash and Das (2012) and Date et al. (2019). ^+^Effect of Hg contamination assessed by analysis of variance (ANOVA); significant values (*P* < 0.05) are in bold. ^#^Alignment against nucleotide sequences containing primer-binding site (see Material & Methods).

**FIGURE 3:**
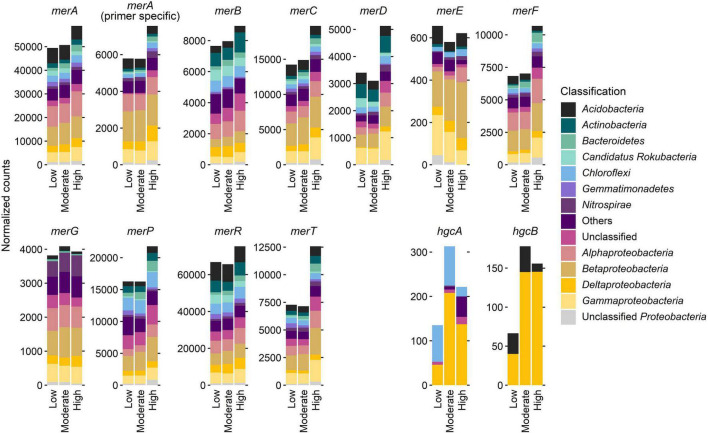
Normalized counts of *mer* and *hgc* genes at the three sites with different Hg contamination levels (low, moderate, high), and itemized according to the most dominant bacterial phyla or subphyla. Whereas *Alpha*-, *Beta*-, and *Gammaproteobacteria* belong to the phylum *Proteobacteria*, *Deltaproteobacteria* now belong to the novel phylum-level lineage *Thermodesulfobacteriota* and *Myxococcota* (Oren and Garrity, 2021).

Normalized counts of *mer* and *hgc* genes at the sites with different Hg contamination levels, itemized according to the most dominant bacterial phyla or subphyla, revealed a dominant role of the phylum *Proteobacteria* (syn. *Pseudomonadota*) and its subphyla *Alpha*-, *Beta*-, *Gamma*-, and *Deltaproteobacteria* (Figure 3). In particular, most counts for almost all *mer* genes were found in *Betaproteobacteria*. Exceptions are *merB* genes, which are most abundant in *Alphaproteobacteria*, and *merD* genes, which are most abundant in *Gammaproteobacteria*. In contrast, *hgc* genes (*hgcA, hgcB*) are most abundant in *Deltaproteobacteria*. However, the *Deltaproteobacteria* have recently been classified into the new phylum-like lineages *Thermodesulfobacteriota* and *Myxococcota* (Oren and Garrity, 2021).

**FIGURE 4:**
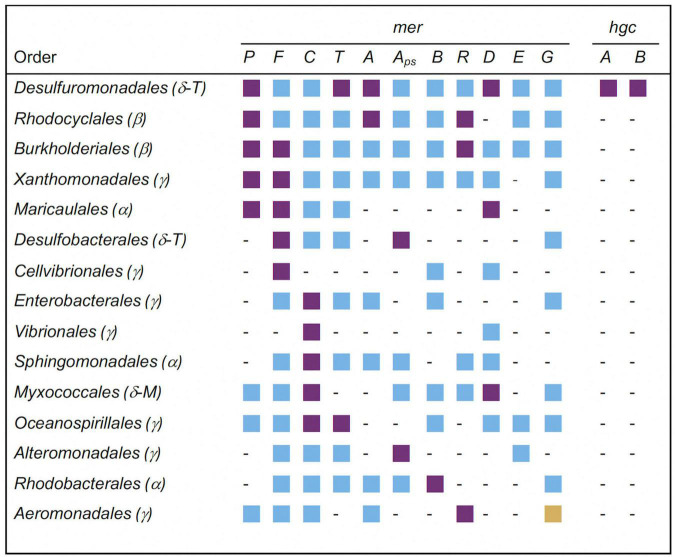
Significantly overrepresented orders of the phylum *Proteobacteria* (syn. *Pseudomonadota*; subphyla: *Alpha(α)-, Beta(β)-, Gamma(γ)-, Delta(δ)proteobacteria*) carrying *mer*- and *hgc*-genes in soil with the highest Hg contamination level. *Deltaproteobacteria* are currently placed in the novel phylum-level lineages *Thermodesulfobacteriota* (*T*) and *Myxococcota* (*M*) (Oren and Garrity, 2021). *Mer* and *hgc* genes correspond to those listed in Table 2. *A*_*ps*_: *merA*_primer specific: Alignment against nucleotide sequences containing primer-binding site (see Material & Methods). Purple squares: significantly overrepresented (*P* < 0.05); blue squares: not significantly overrepresented (*P* ≥ 0.05); yellow squares: significantly underrepresented (*P* < 0.05); -: not present.

Significantly overrepresented orders of the *Proteobacteria* carrying *mer* and *hgc* genes in soils with the highest Hg contamination level are displayed in Figure 4. From the 15 listed orders, the most significantly overrepresented *mer* genes were found in the *Desulfuromonadales* (*merP, merT, merA, merD*). The *Desulfuromonadales* were also the only taxa having significantly overrepresented *hgc* genes (*hgcA, hgcB*), with the genus *Geobacter* carrying mainly *hgcA* and the genus *Desulfuromonas* carrying mainly *hgcB* genes (data not shown). Three significantly overrepresented *mer* genes were found in *Rhodocyclales* (*merP, merA, merR*), *Burkholderiales* (*merP, merF*, *merR*) and *Maricaucales* (*merP, merF, merD*). All other orders had only one or two overrepresented *mer* genes (Figure 4).

## Discussion

### Microbial physiological processes under high Hg contamination

Mercury has been reported to have deleterious effects on microorganisms in short-term incubation experiments (a few weeks or months), leading to shifts in soil microbial diversity and community structure (Frey and Rieder, 2013; Frossard et al., 2017). Studies on long-term exposure (> 10 years) to Hg are relatively rare, but recent studies have indicated that it has strong impacts on soil bacterial diversity and community structure (Liu et al., 2014, 2018; Frossard et al., 2018). In the present study, we found that Hg contamination over a period of >80 years had a positive effect on the microbial biomass (DNA content) and bacterial abundance in soils from an agricultural floodplain. This result is in contrast to findings from a previous long-term field experiment where a negative effect of Hg pollution on bacterial abundance was found (Liu et al., 2018). The soils examined by Liu et al. (2018) came from Hg-mining areas in China that had been heavily contaminated with Hg and MeHg for more than 600 years. The Hg concentrations in the highly contaminated site in our study were approximately 36,000 μg Hg kg^–1^ soil, which is in the range of the concentrations studied by Liu et al. (2018). However, the soils investigated by Liu et al. (2018) also contained high concentrations of MeHg, with values of up to 8 μg MeHg kg^–1^. We did not measure MeHg concentrations in the present investigation, but earlier studies from the same highly contaminated area reported lower values of up to 2 μg MeHg kg^–1^ soil and a MeHg:Hg ratio of 0.15% (Gygax et al., 2019).

In our study, the soluble Hg fraction was relatively low compared to the total Hg concentrations, which can partly be explained by the high soil pH (around 8.0). In general, the solubility of Hg^2+^ decreases in soils with pH >7.0 and higher contents of clay (Frey and Rieder, 2013; Frossard et al., 2017). We assume that a major part of Hg^2+^ was adsorbed on the soil matrix. However, the solubility of Hg^2+^ was considerably higher in the Hg contaminated sites (Table 1). Soluble Hg was about 10 times higher in the high contaminated site than in the moderate and about 35 times higher than in the low contaminated site. We therefore assume that a combination of low solubility of Hg in soils and enhanced bacterial cellular mechanisms to detoxify soluble Hg in soils was responsible for the relatively low impact of the high Hg contamination levels on microbial soil functions.

In the present study, we were interested in whether high Hg contamination also affects the functional attributes of microorganisms (e.g., relative abundance of functional genes) in the long term and their biogeochemical potential in processes such as litter decomposition and N cycling. However, despite elevated concentrations of Hg in these soils, microbial gene potential for C and N cycling were only slightly hampered. In particular, genes of the CAZy functional types for lignin, cellulose and hemicellulose degradation were not affected significantly. The only functional type of the CAZy database, which was significantly influenced by increasing Hg contamination levels was pectin degradation.

Surprisingly, no major N-cycling process was found to be negatively affected. In particular, genes related to functions of dissimilatory nitrate reduction and N fixation showed an overall increase in soils with the highest Hg level. However, important functions of the N cycling were not significantly affected by high Hg concentrations, such as assimilatory nitrate reduction, nitrification, denitrification, and organic degradation/synthesis. In contrast, increased Hg levels in soils are known to negatively affect N-cycling microbial communities in the short-term (Liu et al., 2010; Zhou et al., 2012; Zhu et al., 2021). Similarly, in short-term incubation experiments, nitrification driven by ammonia-oxidizers has been shown to be sensitive to heavy metal stress (Frey et al., 2008; Tipping et al., 2010). However, in our study of soils exposed to long-term Hg contamination, neither the potential nitrification activity nor the abundance of ammonia oxidizers, measured as *amoA* gene copies, were negatively affected by Hg. Overall, we provide novel evidence that the microbial gene potential for major C- and N-cycling processes was not affected negatively by high Hg contamination levels in the long term.

The relatively low responsiveness of the functional attributes of C- and N-cycling to the higher Hg concentrations in soils may be connected to the sorption of Hg^2+^ to iron oxides and organic matter in the soil (Frey and Rieder, 2013). The tolerance of soil microbial communities to Hg mainly depends on the Hg solubility in the soil (Frossard et al., 2017), which is directly influenced by the physical and chemical properties of the soil. In short-term experiments, Frey and Rieder (2013) reported a critical limit concentration for soluble Hg of 0.004 μg Hg kg^–1^ soil (4 mg Hg kg^–1^), which is considerable higher than the water-extractable Hg concentration of 70 μg Hg kg^–1^) found at our study site (Frossard et al., 2018).

The unchanged microbial functionality observed here means that other bacterial groups have taken over the functions that maintain biogeochemical cycling, i.e. there is functional redundancy. This was also confirmed with physiological parameters, such a basal respiration and nitrification rate, which were not significantly altered by the higher Hg levels. The soil biota has a unique capacity to resist events that cause disturbance or change, and some ability to recover from these perturbations (Tibbett et al., 2020). Microbial functional redundancy, where certain taxa contribute equally to certain functions so that one taxon can replaces another, is often considerable in soils (Louca et al., 2017, 2018). In our study, it seems that the replacement of Hg-sensitive taxa with functionally similar but Hg-tolerant taxa prevented a loss of soil functions (Girvan et al., 2005; Tobor-Kaplon et al., 2005). From a functional perspective, our analysis demonstrates that taxonomic turnover along the Hg contamination gradient was coupled with compensation of the functional attributes (i.e. contribution to functional divergence) of the dominant species that are considered as typical drivers of ecosystem processes (Chen et al., 2022). Our results indicate that the lack of effect of long-term Hg contamination on C- and N-cycling processes is likely due to compensatory processes in which sensitive taxa are replaced by functionally similar but tolerant taxa (Griffiths and Philippot, 2013; Liess et al., 2017).

The toxicity of Hg, among other heavy metals, means it has deleterious effects on microorganisms, causing protein denaturation, cell envelope disruption, inhibition of cell division and enzyme activities, DNA damage, and transcriptional inhibition (Nies, 1999; Lemire et al., 2013; Chen et al., 2018b). We therefore wanted to understand how soil microorganisms adapt to high Hg levels in soils, as Hg-tolerant taxa must have tolerance or detoxification coping mechanisms. For this purpose, we evaluated the relationship between Hg contamination and functional genes of Hg detoxification and transformation (e.g., inorganic ion transport and metabolism). Functional characterization via COG analysis revealed significant differences in the genes involved in these processes between soils with low and high Hg concentrations, indicating that microbial communities have the capacity to repair some of these cellular damages caused by Hg. Notably, the relative abundance of metabolic genes related to ion transport, such as inorganic ion transport and metabolism (P), lipid transport and metabolism (I), and coenzyme transport and metabolism (H), were significantly increased under higher Hg concentrations. Similarly, metabolic genes in cluster II related to cellular processes such as cell wall/membrane/envelope (M), intracellular trafficking, secretion, and vesicular transport (U) and posttranslational modification (O), were significantly enhanced in soils with higher Hg concentrations. In its oxidized and monomethyl form, Hg has a strong affinity for the sulfur atom of cysteine (Barkay et al., 2003; Nies, 2003) and interferes with protein structure and function (Møller et al., 2014). In our study, this was reflected in the higher abundance of genes associated with DNA replication and repair (e.g., replication, recombination and repair inorganic) and with sulfur metabolism (inorganic ion transport and metabolism) to create sulfur starvation conditions for Hg tolerance (Hsu-Kim et al., 2013). Furthermore, the relative abundance of genes involved in membrane proteins tended to increase with higher Hg levels, suggesting that Hg stress may stimulate the enzyme activities responsible for Hg transportation through the cell membrane (Barkay and Wagner-Döbler, 2005; Liu et al., 2018).

We also observed a significant increase in the relative abundances of genes relevant to Hg transport (i.e., Hg transport proteins). Previous studies have suggested that inorganic Hg^2+^ can be transported into microbial cells, probably through Hg transport proteins (Schaefer et al., 2011) and the cellular Hg^2+^ is subsequently methylated to highly neurotoxic MeHg by the methylating HgcAB proteins (Parks et al., 2013). Overall, these gene predictors are associated with Hg transformations, which are important biomarkers of soil Hg pollution. Moreover, our study shows that the soil microbial community is able to maintain intracellular homeostasis of essential heavy metals even at high levels of Hg contamination and to normalize resistance to the toxic, non-essential heavy metal Hg. This is probably due to various resistance mechanisms through chromosomal/plasmid-mediated efflux systems that pump the toxic metal ions out of the microbial cells, through the enzymatic biotransformation of metals to produce less toxic Hg-specimens, or through incorporation of Hg into complexes by Hg-binding proteins, making Hg less toxic to the cells (Nies, 1999; Hsu-Kim et al., 2013; Chen et al., 2018a).

### Microbial cellular mechanisms to detoxify Hg

The relatively small effect of the high Hg contamination levels on the functional attributes of the C- and N-cycling genes (see above) might be attributed to the efficient Hg detoxification system of the microbial community. The high Hg concentrations may be connected to the conversion of Hg^2+^ to elemental Hg (Hg^0^) via the activities of members of the microbial community possessing the Hg-resistant *mer* gene (Barkay et al., 2003). This is because Hg^0^ evaporates from the cells of Hg-resistant microorganisms. Therefore, we looked specifically at the *mer* operon carrying a number of genes and gene products closely related to Hg tolerance and the reduction mechanism of these bacteria (Barkay et al., 2003). The *mer* determinants are classified into two types: a narrow-spectrum one that detoxifies only inorganic Hg through the main *mer*A gene and broad-spectrum one that detoxifies both organic and inorganic Hg through *mer*A and *mer*B genes (Boyd and Barkay, 2012). The *mer* operon is composed of the operator, promoter, regulator genes, and functional genes such as *mer*A to *mer*G, *mer*P, *mer*R, and *mer*T. All these genes code for different proteins that participate in the detection, scavenging, transport and reduction of Hg (Barkay et al., 2003; Lin et al., 2011). At its core, the *mer* operon encodes a homodimeric flavin-dependent disulfide oxidoreductase, termed Hg reductase enzyme encoded by *merA*, that functions to reduce Hg^2+^ to volatile Hg^0^ leading to removal of the metal by passively diffuses out of the cell (Boyd and Barkay, 2012). In our soils, the *merA* genes were slightly (but significantly in the primer-specific analysis) enhanced under the highest Hg concentrations. In fact, the quantitative PCR analysis of *merA* genes was somewhat in accordance with the results from shotgun metagenomics. The augmentation of *merA* gene copy numbers in the highest Hg contaminated soils suggests the occurrence of Hg resistance, which might be due either to a transfer of this mobile genetic element among bacteria, induced by the presence of Hg in the soil, or to a species sorting process favoring bacterial cells containing the *merA* gene (Frossard et al., 2018). Horizontal gene transfer of *merA* genes has been shown to be enhanced in the presence of Hg (Puglisi et al., 2012).

Furthermore, *merB* genes were significantly increased in the highest Hg contamination levels, indicating that the soil microbiome showed the potential for demethylation of MeHg even with severe contamination. This operon may code for organo-Hg lyase (MerB), which catalyzes the protonolytic cleavage of the C–Hg bond in organo-Hg compounds, among them MeHg, making the Hg ion available for the reduction (Barkay et al., 2003). Our data are also consistent with previous findings that the abundance of *merB* genes was about 10 times lower than that of *merA* and *merR* genes in Hg-contaminated environments, according to metagenomic analysis (Christensen et al., 2019). Hg demethylation is a typical process for alleviating the toxicity of MeHg (Liu et al., 2018). It is mediated by several enzymes, including the alkyl-Hg lyase protein (MerB) that degrades MeHg to Hg^2+^ and subsequently reduced to Hg^0^ by the MerA reductase (Barkay et al., 2003; Zhou et al., 2020). The combinatorial action of *merA* and *merB* allows the complete detoxification of a broad spectrum of Hg compounds, providing a major decontamination mechanism for various microbial lineages in environments contaminated with Hg. In addition to *merA* and *merB*, *mer* operons may code for a periplasmic Hg ion scavenging protein (MerP) and one or more inner membrane-spanning proteins (MerC, MerE, MerF, and MerT), which transport Hg ions to the cytoplasmic MerA protein (Barkay et al., 2003; Lin et al., 2011). Except for *merE* all these functional genes were significantly enhanced in the highest Hg contamination level in our study.

### Bacterial taxa involved in Hg detoxification

We predominantly found *mer* genes in *Acidobacteria*, *Actinobacteria*, *Bacteroidetes*, *Chloroflexi*, *Proteobacteria* and *Nitrospirae*. The widespread taxonomic distribution of *mer* genes is well known (Boyd and Barkay, 2012; Frossard et al., 2017; Vigneron et al., 2021) which may explain the wide taxonomic range of Hg-tolerant bacteria found in the Hg contaminated soils. The highest number of normalized counts of *mer* genes were found in *Proteobacteria* in particular *Betaproteobacteria*, consistent with findings reported previously for a gradient of Hg contamination in a short-term incubation experiment (Frey and Rieder, 2013). We observed that *merP*, a gene encoding a periplasmatic Hg-ion-binding protein, was increased in soils with the highest Hg concentration. *merP* was found in *Desulfuromonadales*, *Rhodocyclales*, *Burkholderiales*, *Xanthomonadales* and *Maricaulales*, all known to contain a periplasmic Hg-ion-scavenging protein (MerP), which transports Hg ions to the cytoplasm transporter proteins, which in turn enable Hg to enter the cytoplasm (Lin et al., 2011).

Normalized counts of *HgcAB* genes detected in this study were low compared to *mer* genes. Hg methylators often constitute a small proportion of the microbiome in oxic soils (Schaefer et al., 2014; Bravo and Cosio, 2020). Mercury-methylating microorganisms thrive in oxygen-deficient environments (e.g., rice paddies, wetlands, sediments, anoxic waters) in which redox conditions play an important regulating role for both the activity of Hg^2+^ methylating microorganisms and the availability of Hg^2+^ for methylation (Podar et al., 2015). MeHg concentrations were relatively low (up to 2 μg MeHg kg^–1^ or MeHg:Hg ratio of 0.15 %) in the soils considered here (Gygax et al., 2019), and therefore we expect that Hg methylation is low in these relatively sandy soils (sand content: 23–44%). Microorganisms are the primary driver of Hg methylation through the activity of a corrinoid protein, *hgcA*, and a ferredoxin, *hgcB* (Parks et al., 2013; Podar et al., 2015). We found that the functional gene *hgcB* was enriched in soils with the highest Hg concentration. *HgcAB* genes were most abundant in *Deltaproteobacteria*, suggesting that *Deltaproteobacteria* were likely the primary Hg methylators at this site. The capacity to perform Hg methylation was historically associated with certain sulfate- and iron-reducing *Deltaproteobacteria* and methanogenic *Archaea* (Gilmour et al., 2013; Bravo and Cosio, 2020; Capo et al., 2020). The *hgcAB* genes that were detected were mainly found in the order of *Desulfuromonadales*. Members of *Desulfuromonadales* are known to be potential methylating organisms (Bravo et al., 2018a,b; Xu et al., 2019). The diversity of known Hg methylators is expanding, however, as increasing numbers of *hgcAB* sequences are being identified from shotgun metagenomics, metagenome-assembled genomes (MAGS), and *hgcAB* amplicon-specific sequencing (Jones et al., 2019; McDaniel et al., 2020).

Interestingly, in our study, the relative abundance of the phylum *Nitrospirota*, including the genus *Nitrospira*, based on 16S rRNA genes retrieved from the metagenomes, did not change along the Hg contamination gradient. This result was also confirmed by our recent amplicon sequencing dataset from the same field experiment (Frossard et al., 2018). Members of *Nitrospirota* (i.e. *Nitrospirae*) contained Hg detoxification genes (Figure 3) and were therefore tolerant to Hg. We therefore assume that *Nitrospirota* were not affected by Hg contamination, which is in contrast to a recent study of Mahbub et al. (2020) showing that *Nitrospirae* were sensitive to higher Hg levels in soils and did not recover after four years of exposition. We therefore concluded that this could be the reason why the nitrification process and the genes involved in it were not disturbed in our study.

## Conclusion

Our shotgun metagenomic study of microbial communities of soils contaminated with low to high levels of Hg for over 80 years demonstrated that microbial processes relevant for C and N cycling were not significantly affected by higher levels of Hg contamination. This is particularly the case for the microbial gene potential for cellulose and lignin degradation, assimilatory nitrate reduction, denitrification, nitrification, organic degradation or organic synthesis. Although we observed a significant change in the functional beta-diversity of the predicted microbial genes with long-term Hg contamination, a shift in the functional capabilities of the microbial communities was not obvious. This means that Hg-tolerant microbial taxa have taken over the functions that maintain biogeochemical cycling. This process can be considered functional redundancy, a property that is often observed in microbial soil communities but was not expected here after long-term exposure to high Hg levels. It therefore seems that microbial communities can withstand considerable Hg stress and can even detoxify Hg. A significant increase in the Hg-detoxifying *mer* and *hgc* genes was observed, with the overrepresented genes being able to bind Hg ions, to transport them across membranes, to methylate and demethylate them, and to reduce them to volatile Hg. Overall, we conclude that long-term exposure to high Hg contamination is not harmful to the microbial community. Although we see shifts in the functional beta-diversity of the predicted microbial genes, we find functional redundancy rather than a dramatic change or breakdown in functional capabilities.

## Data availability statement

The datasets presented in this study can be found in online repositories. The names of the repository/repositories and accession number(s) can be found below: https://www.ncbi.nlm.nih.gov/, bioproject/PRJNA794054.

## Author contributions

BF and IB designed the microbial study and wrote the main parts of the manuscript. WQ and BS performed genetic analyses in the lab. BF, BMR, and IB performed statistical analyses. All authors contributed to the final version of the manuscript.

## Footnotes

1
https://www.bioinformatics.babraham.ac.uk/projects/fastqc/
