# Supplementary material for: Long-term mercury contamination does not affect the microbial gene potential for C and N cycling in soils but enhances detoxification gene abundance
Source: Front Microbiol. 2022 Oct 5;13:1034138. doi: 10.3389/fmicb.2022.1034138 (PMC9581213; doi:10.3389/fmicb.2022.1034138)
Supplement: Supplementary file 1 [file Data_Sheet_1.docx]

**Supplementary Material**

**Long-term mercury contamination does not affect the microbial gene potential for C and N cycling in soils but enhances detoxification gene abundance**

**Beat Frey^1†^*, Basil M. Rast^1†^, Weihong Qi^2^, Beat Stierli^1^, Ivano Brunner^1^**

**^1^** Forest Soils and Biogeochemistry, Swiss Federal Institute for Forest, Snow and Landscape Research WSL, CH-8903 Birmensdorf, Switzerland

**^2^** Functional Genomics Center Zürich FGCZ, ETH Zürich, CH-8057 Zürich, Switzerland

***Corresponding author**: Dr. Beat Frey, Swiss Federal Institute for Forest, Snow and

Landscape Research WSL, CH-8903 Birmensdorf, Switzerland, Email: beat.frey@wsl.ch, Phone: +41 44 739 25 41

**†** These authors have contributed equally to this work

Supplementary Tables: 3

Supplementary Figure: 1

**Table 1.** Statistics of the genome assembly.

**Parameter Size**

Assembly size (bp^+^) 12,375,076,959

Number of high-quality reads after filtering 786,878,084

Number of MEGAHIT assembly of reads into contigs 17,743,321

Maximum contig length (bp) 207,300

Minimum contig length (bp) 200

Mean contig length (bp) 697

N_50_ contig length (bp) 782

Number of predicted protein-coding genes with MetaGeneMark^†^ 26,237,952

Number of predicted protein-coding genes annotated with EggNOG database^*^ 12,468,915

Number of predicted protein-coding genes annotated with CAZy database^#^ 262,248

Number of predicted protein-coding genes annotated with NCyc database^‡^ 42,859

^+^ bp: base pairs

^†^ MetaGeneMark: Tool for gene prediction in metagenomes, utilizing metagenome parameters and gene prediction

^*^ EggNOG: Database of evolutionary genealogy of genes: non-supervised orthologous groups

^#^ CAZy: Carbohydrate-active enzymes database

^‡^ NCyc: Curated integrative database for fast and accurate metagenomic profiling of N cycling genes

**Table 2.** Richness and Shannon index of normalized counts for all predicted genes and for genes of the EggNOG, CAZy, and NCyc databases.

**Richness Shannon**

**Database Low Hg Moderate Hg High Hg Low Hg Moderate Hg High Hg**

All predicted genes 12,063,152 12,493,639 11,582,793 15.6 15.8 15.7

EggNOG 6,846,271 7,099,416 6,594,610 15.0 15.2 15.1

CAZy 183,586 190,025 177,268 11.3 11.5 11.4

NCyc 28,109 29,496 28,146 9.4 9.7 9.6

**Table 3.** Main effects on functional gene alpha-diversity (Richness and Shannon index) and beta-diversity of all predicted genes and the genes annotated with the EggNOG, CAZy, and NCyc databases for soils with different Hg contamination levels (low, moderate, high).

**Alpha-diversity***

**Richness Shannon Beta-diversity^+^**

**Databases *DF*^#^ *F P F P* Pseudo-*F* *P***

All predicted genes 2 2.98 0.42 3.06 0.16 3.08 **0.011**

EggNOG 2 3.01 0.43 3.21 0.15 2.76 **0.016**

CAZy 2 2.93 0.48 3.11 0.17 3.03 **0.013**

NCyc 2 3.10 0.39 3.30 0.09 2.72 **0.015**

* Effect of Hg contamination assessed by analysis of variance (ANOVA).

^+^ Effect of Hg contamination assessed by permutational multivariate analysis of variance (PERMANOVA).

^#^ Values represent degrees of freedom (*DF*), *F*-ratio (*F*), pseudo-*F* ratio (Pseudo-*F*), and the level of significance (*P*); significant values (*P* < 0.05) are in bold.


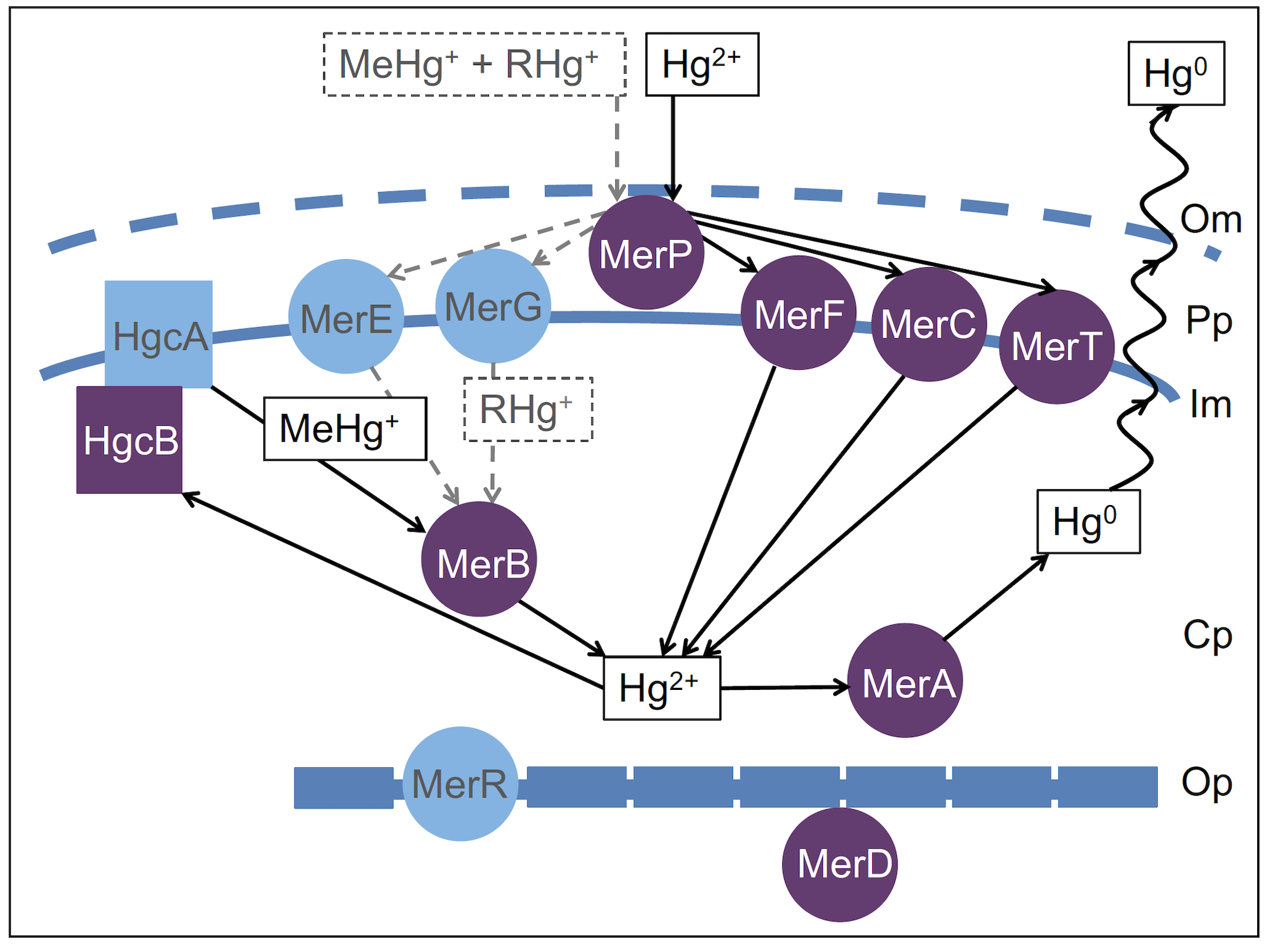


**Figure 1:** Schematic pathways of Hg^2+^, MeHg^+^, RHg^+^, and Hg^0^, and the location and functions of MerA to MerG, MerP, MerR, and MerT proteins (circles) and of HgcA and HgcB proteins (squares) in a bacterial cell, following the schemes of Dash et al. (2012) and Date et al. (2019). Purple shading indicates proteins of significantly overrepresented genes at the high Hg contamination level, as indicated in Table 9. Cp: cytoplasm, Im: inner membrane, MeHg^+^: methyl Hg, Om: outer membrane, Op: *mer* operon, Pp: periplasm, RHg^+^: organic form of Hg.
